# The complete chloroplast genome of *Geum longifolium* (Maxim.) Smedmark 2006 (Rosaceae: Colurieae) and its phylogenomic implications

**DOI:** 10.1080/23802359.2023.2270212

**Published:** 2023-10-18

**Authors:** Jia-Jie Guo, Zhi-Ping Zhang, Khasbagan, Soyolt, Qin-Qin Li

**Affiliations:** a College of Life Science and Technology, Inner Mongolia Normal University, Hohhot, China; b Key Laboratory of Biodiversity Conservation and Sustainable Utilization for College and University of Inner Mongolia, Hohhot, China; c College of Computer Science and Technology, Inner Mongolia Normal University, Hohhot, China

**Keywords:** Chloroplast genome, Colurieae, *Geum longifolium*, Rosaceae

## Abstract

*Geum longifolium* (Maxim.) Smedmark 2006 belongs to the family Rosaceae, subfamily Rosoideae, tribe Colurieae. *Geum longifolium* is endemic to China and its whole herb is used in Chinese medicine. Here*,* the first complete chloroplast (cp) genome of *G. longifolium* was assembled and annotated based on genome skimming, and its phylogenetic position was investigated using phylogenomic evidence. The cp genome size of *G. longifolium* was 155,884 bp with the total GC content of 36.7%. Its cp genome presented a typical tetrad structure, composed of a large single copy (LSC) region (85,338 bp), a small single copy (SSC) region (18,358 bp), and a pair of inverted repeat (IR) regions (26,094 bp). The cp genome encoded 129 genes, including 84 protein-coding genes, 37 tRNA genes, and eight rRNA genes. Phylogenetic analysis indicated that *G. longifolium* was sister to *G. elatum* Wall. ex G.Don 1832 in current taxa sampling. This study can enrich the chloroplast genomic resource of *Geum* and lay the foundation for future phylogenetic studies on *Geum*.

## Introduction

**Figure 1.**
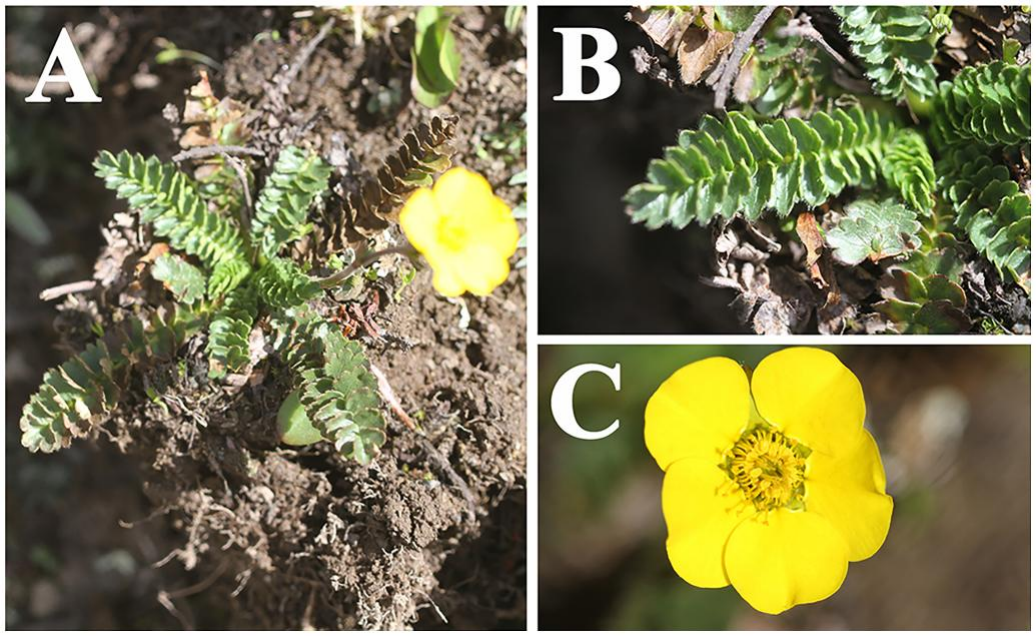
Species reference image of *Geum longifolium* in this study. (A) whole plant; (B) basal leaf; (C) flower. Species images were taken by the corresponding author Qin-Qin Li in Qilian county, Qinghai province, China.

*Geum longifolium* (Maxim.) Smedmark 2006 (synonym: *Coluria longifolia* Maxim. 1882; Figure 1) is a member of the family Rosaceae Juss., subfamily Rosoideae (Juss.) Arn., tribe Colurieae Rydb. (Smedmark 2006). *Geum longifolium* is endemic to China and distributed in the alpine meadows of Gansu, Qinghai, Sichuan, Xizang, and Yunnan (Li et al. 2003). Whole herb of *G. longifolium* is used as medicine with the effect of hemostasis, pain relief, and heat-clearing (Yü and Kuan 1985). The chloroplast (cp) genome of *G. longifolium* has not been reported to date and its phylogenetic position has not been investigated using the phylogenomic evidence. In the present study, we reported the complete cp genome of *G. longifolium* for the first time and inferred its phylogenetic relationships with related *Geum* species. Our study can make a great contribution to further studies on the taxonomy, phylogeny, and population genetics of *Geum* species.

## Materials

Leaf sample of *G. longifolium* was collected from Qilian County, Qinghai Province, China (38°01.395' N, 100°14.278' E). The specimen was deposited at the herbarium of Inner Mongolia Normal University (NMTC) (http://bio.imnu.edu.cn, Qin-Qin Li, liqq@imnu.edu.cn) under the voucher number Li QQ BDB1.

## Methods

Plant total DNA was extracted from silica gel-dried leaf by modified Cetyl Trimethyl Ammonium Bromide (CTAB) method (Doyle and Doyle 1987). Subsequently, the prepared DNA library with an insert size of 300 bp fragments was sequenced by the Illumina NovaSeq 6000 platform in Novogene (Beijing, China). Trimmomatic version 0.33 (Bolger et al. 2014) was used to remove adapters after sequencing and a total of 40,106,684-bp raw reads were obtained. The raw reads were assembled by NOVOPlasty version 3.8.3 (Dierckxsens et al. 2017), with the cp genome of *Geum macrophyllum* Willd.1809 (GenBank accession number MT774132; Li and Wen 2021) as the reference sequence and its ribulose-1, 5-bisphosphate carboxylase/oxygenase (*rbcL*) gene as the seed and 3,655,282-bp reads were mapped to *G. macrophyllum* cp genome. Sequencing depth and coverage map of *G. longifolium* was generated following the protocol of Ni et al. (2023). The cp genome annotation of *G. longifolium* was conducted using transferring annotations by Geneious prime (Kearse et al. 2012), with the cp genome of *G. macrophyllum* (MT774132) as the reference. Chloroplast Genome Viewer (CPGView) was used to draw the circular cp genome map of *G. longifolium* and the structure of the genes that are difficult to annotate in the cp genome (Liu et al. 2023).

To infer the phylogenetic position of *G. longifolium,* we conducted a phylogenetic analysis of* G. longifolium* and its related species. Sixteen cp genome sequences were downloaded from GenBank, including 12 Colurieae accessions and four other Rosoideae species. Based on a previous study (Zhang et al. 2017), we selected four Rosoideae species (*Agrimonia nipponica* Koidz.1930,* Potentilla suavis* Soják 2008,* Rosa multiflora* Thunb 1784, and* Rubus niveus* Thunb.1813*)* as outgroups. The cp genome sequences of the above 17 accessions were aligned using MAFFT version 7.450 (Katoh and Standley 2013) under “auto”settings*.* Software trimAL version 1.4 (Capella-Gutiérrez et al. 2009) was then used to trim the alignment properly with a 0.9 gap threshold. Maximum likelihood (ML) method and Bayesian inference (BI) were used to establish phylogenetic trees respectively. The ML analysis was performed using RAxML version 8 (Stamatakis 2014) following Zhang et al. (2020). The BI analysis was conducted using MrBayes ver. 3.2.2 (Ronquist et al. 2012) following Tian et al. (2020*)* and the GTR + I + G model was selected as the best-fit model by PartitionFinder2 (Lanfear et al. 2017).

## Results

**Figure 2.**
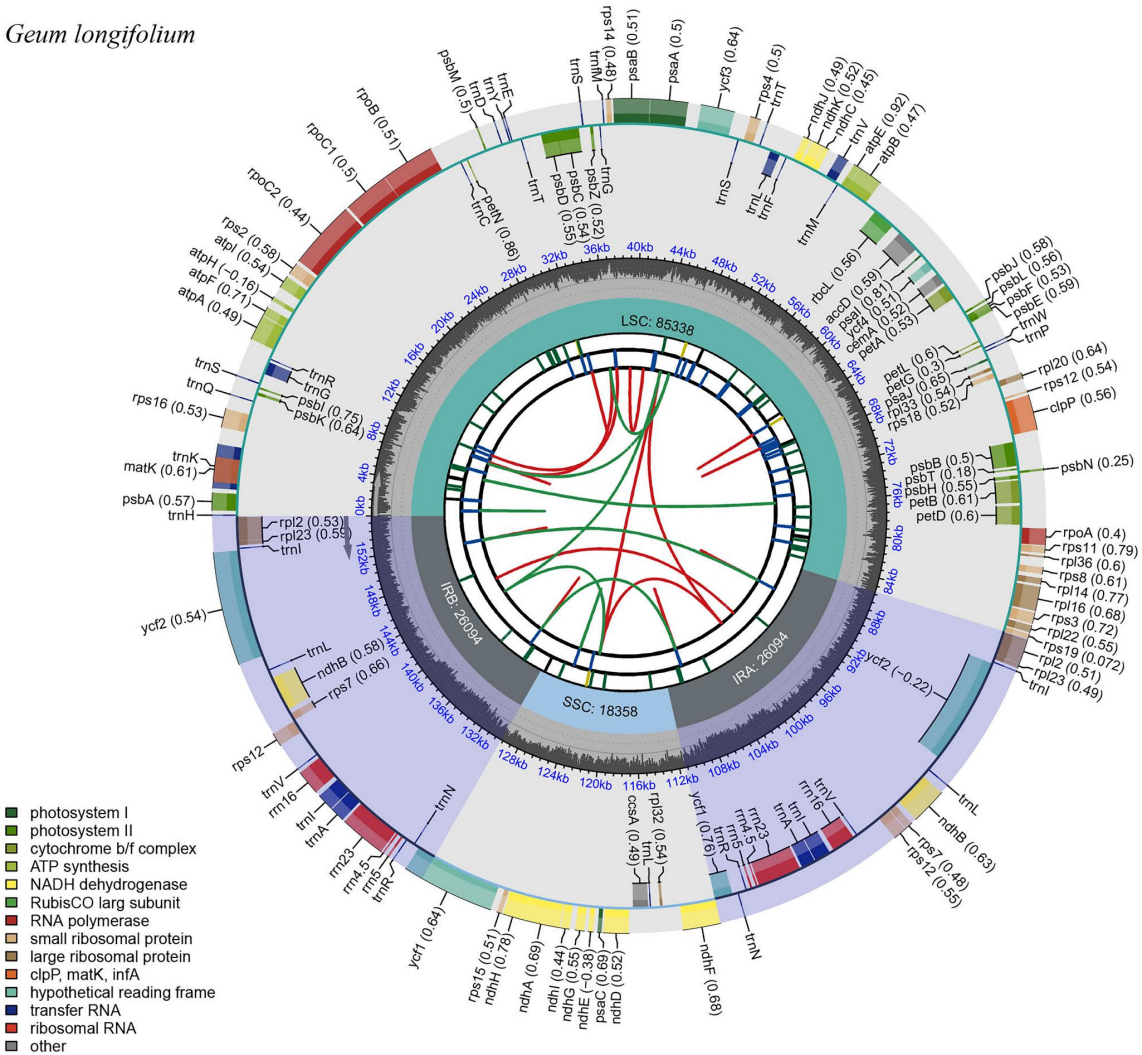
Genome map of *G. longifolium* chloroplast genome drawn by Chloroplast Genome Viewer (CPGView, http://www.1kmpg.cn/cpgview). The genome map includes six tracks. From the inward to outward, the first track shows the dispersed repeats which consist of direct repeats and palindromic repeats, connected with red and green arcs. The second track shows the long tandem repeats (blue bars). The third track shows the short tandem repeats or microsatellite sequences as short bars. The fourth track shows the large single copy (LSC), the small single copy (SSC), and inverted repeat (IRa and IRb) regions. The fifth track shows the GC contents along the chloroplast genome. The outermost track shows the genes which are color-coded based on their functional classification. The inner genes are transcribed clockwise, and the outer genes are transcribed anticlockwise.

The cp genome of *G. longifolium* was a circular DNA molecule (GenBank accession number OP161499; Figure 2). The cp genome size of *G. longifolium* was 155,884 bp, with an average depth of 3697.14 × (Figure S1), and the total GC content was 36.7%. Its cp genome presented a typical tetrad structure, consisting of a large single copy (LSC) region (85,338 bp), a small single copy (SSC) region (18,358 bp), and a pair of inverted repeat (IR) regions (26,094 bp). The GC content of LSC, SSC, and IR were 34.4%, 30.9%, and 42.6%, respectively. The cp genome contained 129 genes, including 84 protein-coding genes, 37 tRNA genes, and eight rRNA genes. In addition, the cp genome contained one pseudogene *Ψycf1* located in the IRb/SSC junction. Ten unique genes (*clpP*, *ndhA*, *ndhB*, *petB*, *petD*, *rpl2*, *rpl16*, *rpoC1*, *rps16*, *ycf3*) were cis-splicing and *rps*12 was trans-splicing (Figure S2).

**Figure 3.**
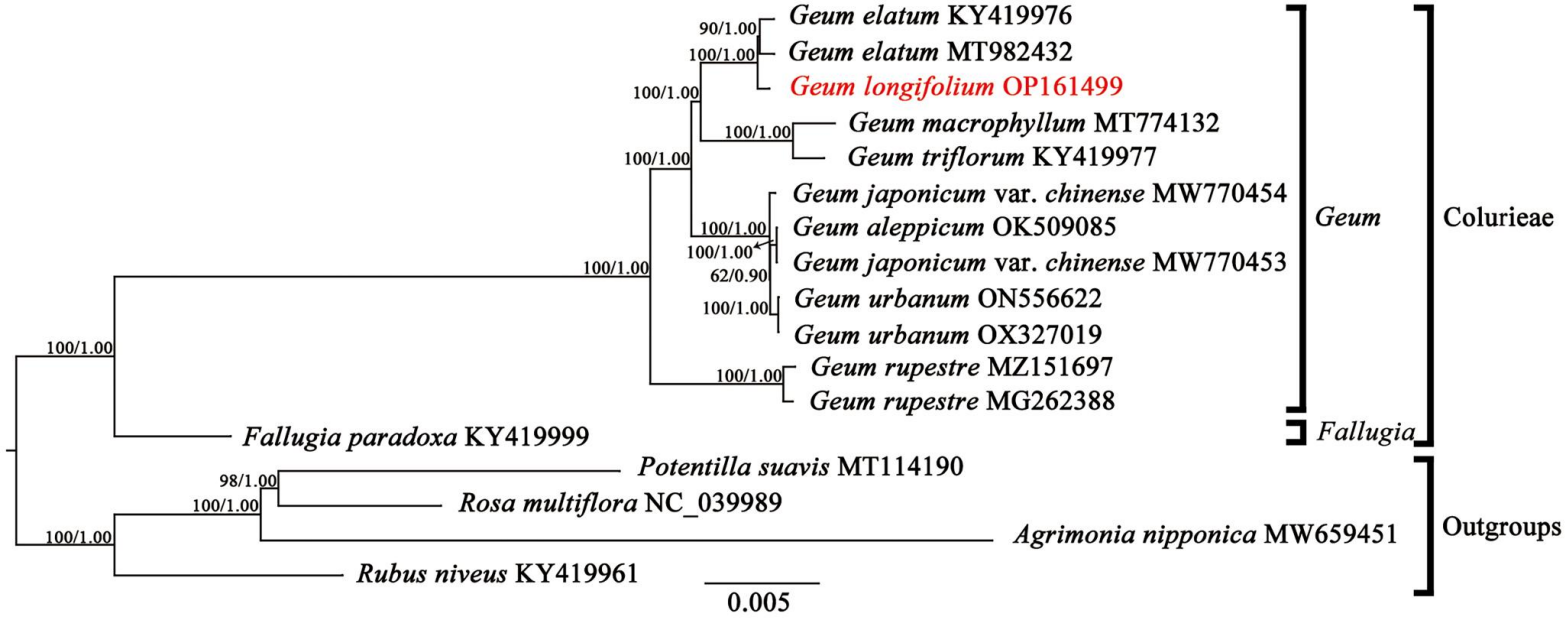
The Maximum-likelihood (ML) phylogenetic tree reconstructed based on 13 cp genome sequences from Colurieae plus four other Rosoideae species as outgroups. Values along branch represent ML bootstrap percentages, and Bayesian posterior probabilities respectively. The following sequences were used: *Geum elatum* KY419976 (Zhang et al. 2017), *Geum elatum* MT982432, *Geum longifolium* OP161499, *Geum macrophyllum* MT774132 (Li and Wen 2021), *Geum triflorum* KY419977 (Zhang et al. 2017), *Geum japonicum* var. *chinense* MW770454, *Geum japonicum* var. *chinense* MW770453, *Geum aleppicum* OK509085 (Zhang *et al*. 2022), *Geum urbanum* ON556622, *Geum urbanum* OX327019, *Geum rupestre* MZ151697, *Geum rupestre* MG262388 (Duan et al. 2018), *fallugia paradoxa* KY419999 (Zhang et al. 2017), *Potentilla suavis* MT114190 (Li et al. 2020), *Rosa multiflora* NC_039989, *Agrimonia nipponica* MW659451, and *Rubus niveus* KY419961 (Zhang et al. 2017).

Tree topologies inferred by ML and BI analyses were identical, so the ML tree with bootstrap support values (BS) and Bayesian posterior probabilities (PP) was shown in Figure 3*.* The genus *Geum* was recovered as a monophyletic group in the phylogenetic tree (BS = 100%, PP = 1.00). Within *Geum*, *G. rupestre* (Yü et Li) Smedmark 2006 is sister to a clade comprising seven other species (*G. urbanum* L. 1753, *G. japonicum* var. *chinense* F.Bolle 1931, *G. aleppicum* Jacq. 1781, *G. triflorum* Pursh 1814, *G. macrophyllum*, *G. longifolium* and *G. elatum* Wall. ex G.Don 1832) in current taxa sampling.

## Discussion and conclusion

In this study, using the related bioinformatics methods, the first complete cp genome of* G. longifolium* was assembled and annotated based on genome skimming. The cp genome of* G. longifolium* has similar structure and gene size, and consistent gene composition and gene order to that of other* Geum* species (Duan et al. 2018, Li and Wen 2021, Zhang et al. 2022). Species *Geum longifolium* was first published under the name of *Coluria longifolia* Maxim. (1882: 466) and the name was adopted in the Flora of China by Li et al. (2003). Smedmark (2006) made a recircumscription of* Geum* based on phylogenetic studies of Colurieae (Smedmark and Eriksson 2002, Smedmark et al. 2003), in which *Coluria longifolia* were included in* Geum* as *G. longifolium* (Maxim.) Smedmark. Our phylogenetic analysis showed that* G. longifolium* was nested within the* Geum* species, which supported Smedmark’s taxonomic treatment to place this species within* Geum* (Smedmark 2006)*.* Phylogenetic analysis indicated that *G. longifolium* was sister to *G. elatum* in current taxa sampling. This study can enrich the chloroplast genomic resource of* Geum* and lay the foundation for future phylogenetic studies on *Geum.*

## Ethical approval

No ethical approval is required. *Geum longifolium* is not an endangered or protected plant.

## Supplementary Material

Supplemental Material

Supplemental Material

Supplemental Material

## Data Availability

The genome sequence data supporting the findings of this study are openly available in GenBank of NCBI (https://www.ncbi.nlm.nih.gov/) under the accession number OP161499. The associated BioProject, SRA, and Bio-Sample accession numbers are PRJNA866517, SRR21050029, and SAMN30167531, respectively.
