# Supplementary figures and images for: The complete chloroplast genome of Geum longifolium (Maxim.) Smedmark 2006 (Rosaceae: Colurieae) and its phylogenomic implications
Source: Mitochondrial DNA B Resour. 2023 Oct 18;8(10):1124–7. doi: 10.1080/23802359.2023.2270212 (PMC10586075; doi:10.1080/23802359.2023.2270212)

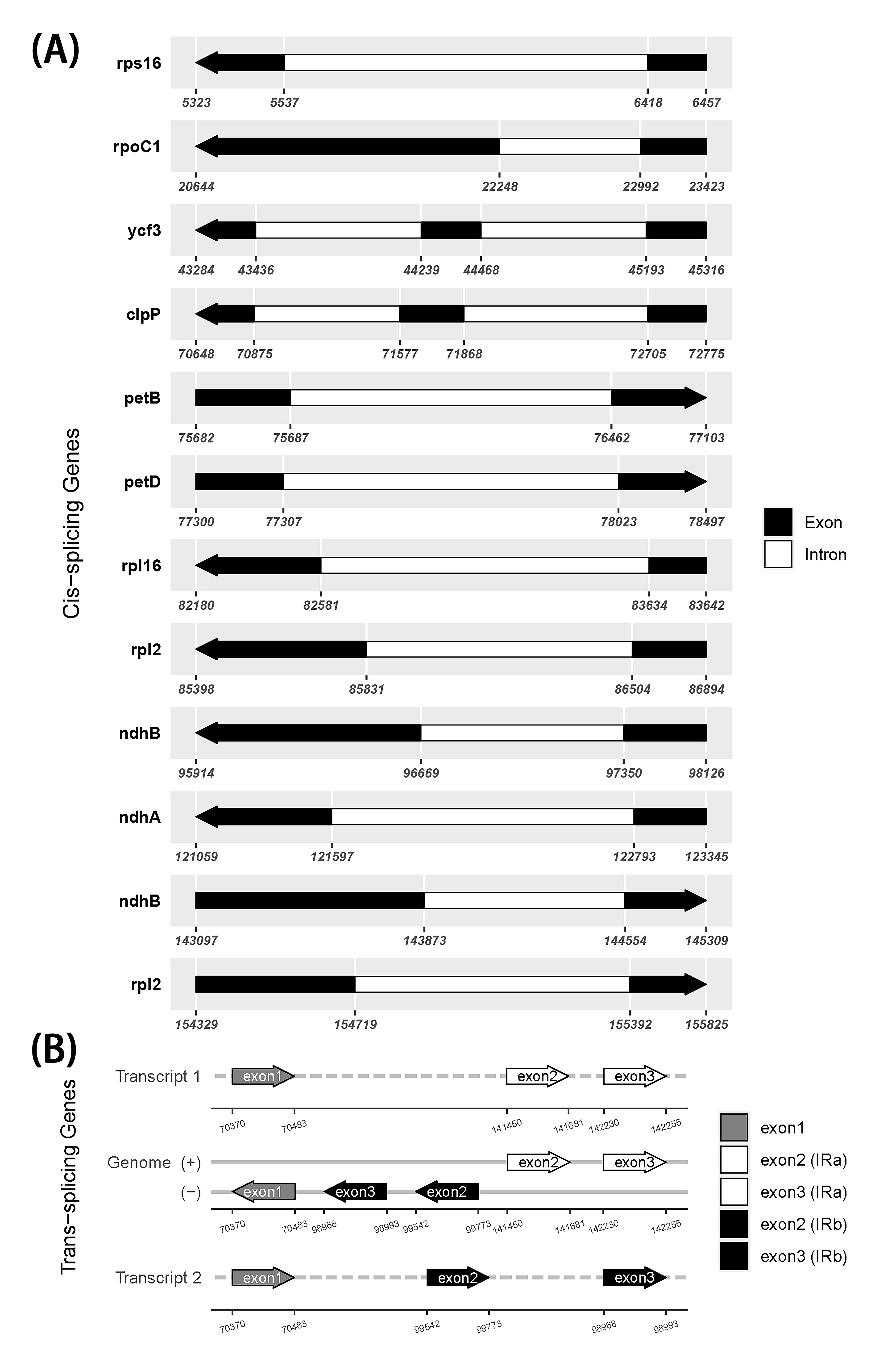

Supplement: Supplemental Material [file TMDN_A_2270212_SM5734.jpg]

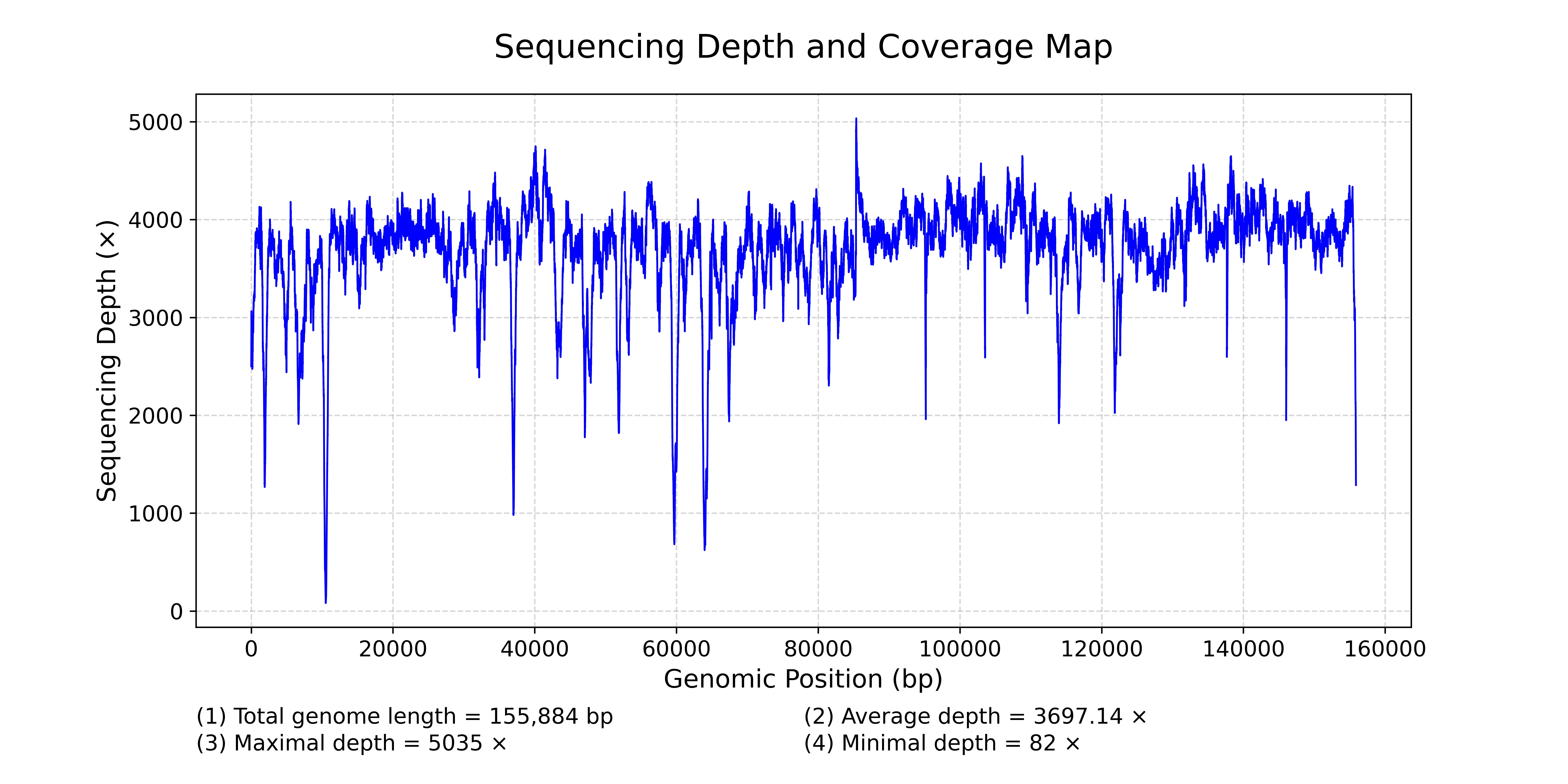

Supplement: Supplemental Material [file TMDN_A_2270212_SM5729.jpg]
