# Supplementary material for: The complete chloroplast genome of Geum longifolium (Maxim.) Smedmark 2006 (Rosaceae: Colurieae) and its phylogenomic implications
Source: Mitochondrial DNA B Resour. 2023 Oct 18;8(10):1124–7. doi: 10.1080/23802359.2023.2270212 (PMC10586075; doi:10.1080/23802359.2023.2270212)
Supplement: Supplemental Material [file TMDN_A_2270212_SM5728.docx]

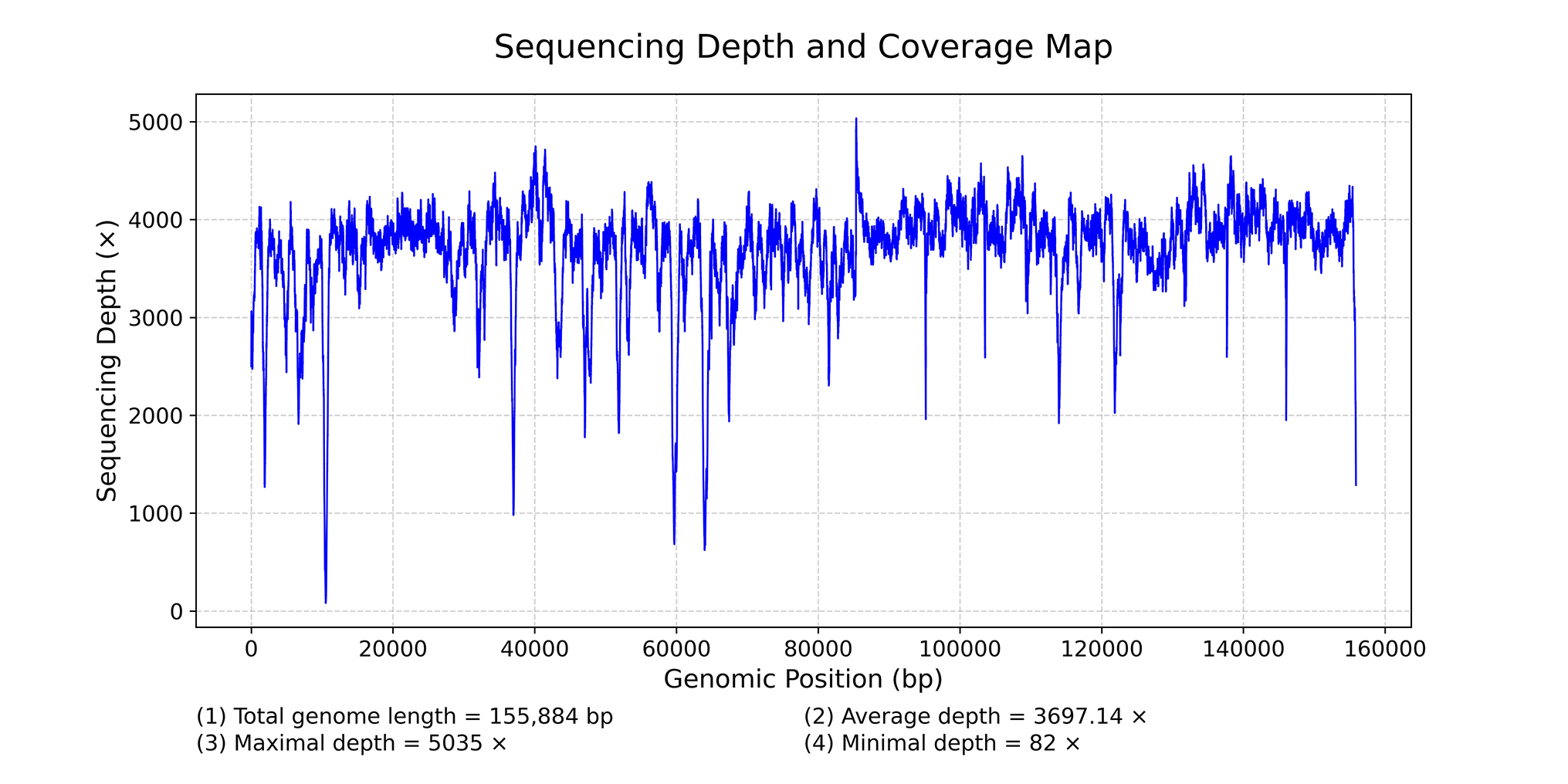


Figure S1. Sequencing depth and coverage map of *Geum longifolium*.


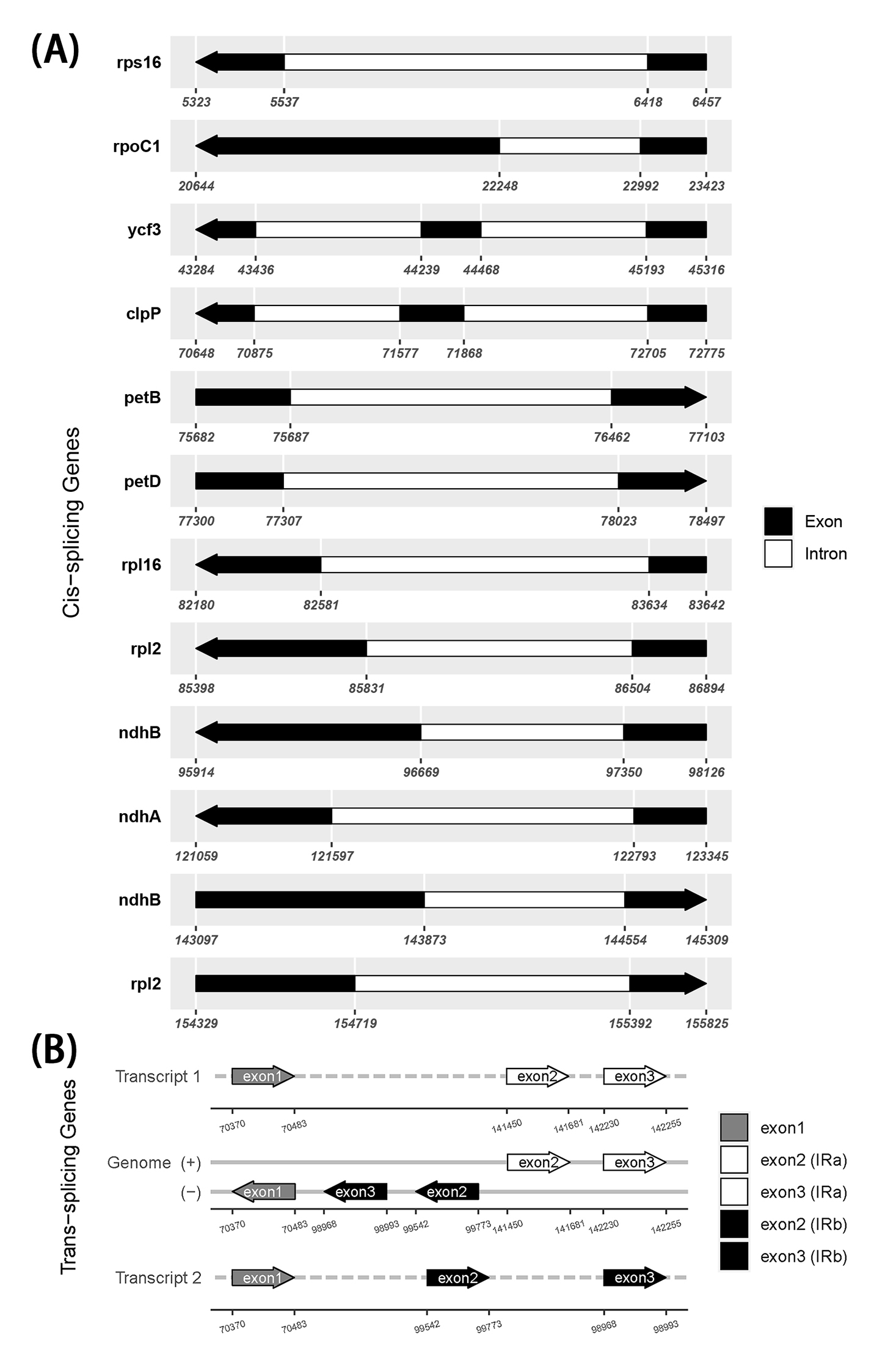


Figure S2. Structure of the genes that are difficult to annotate in the chloroplast genome of *Geum longifolium*. (A) Schematic map of the cis-splicing genes. (B) Schematic map of the trans-splicing gene *rps12*.
